# Arctic Sun Surface Temperature Management Device for Neuroprotection During Pregnancy—A Short Case Report and Review of the Literature

**DOI:** 10.3390/reports8040204

**Published:** 2025-10-16

**Authors:** Vasileios Vazgiourakis, Konstantinos Mantzarlis, Konstantina Deskata, Asimina Valsamaki, Foteini Bardaka, Dimitra Bagka, George Dimopoulos, Demostenes Makris

**Affiliations:** 1 Department of Critical Care, University Hospital of Larissa, Mezourlo, 41110 Larissa, Greece; mantzk@outlook.com (K.M.); konstadv@gmail.com (K.D.); semi_val@hotmail.com (A.V.); bardakafotini@yahoo.gr (F.B.); dbagka@gmail.com (D.B.); appollon7@hotmail.com (D.M.); 2 School of Medicine, University of Thessaly, 41500 Thessaly, Greece; 3 Third Department of Critical Care Medicine, Medical School, National and Kapodistrian University of Athens, 11527 Athens, Greece; gdimop@med.uoa.gr

**Keywords:** hyperpyrexia, neuroprotection, temperature control systems, pregnancy, arctic sun

## Abstract

Targeted temperature management (TTM), particularly the avoidance of hyperpyrexia, is a cornerstone of intensive care, especially in conditions such as cerebral edema and increased intracranial pressure. Management becomes more complex in pregnancy, where maternal neuroprotection must be weighed against fetal safety. Both invasive and noninvasive methods for temperature control have been described, but evidence regarding their safety in pregnancy remains limited. We present the case of a 24-year-old pregnant woman admitted to the ICU with cerebral edema due to subdural empyema. The Arctic Sun surface cooling system was employed for fever control, with continuous maternal and fetal monitoring. The system effectively maintained normothermia without immediate adverse effects on either the mother or the fetus. However, on the third day of its use, the patient experienced a spontaneous preterm delivery of a stillborn fetus. Although a causal link between surface cooling and preterm labor cannot be established from this single case, the event underscores the need for caution and further investigation. This case highlights both the feasibility and the uncertainties of using advanced TTM devices in critically ill pregnant patients. It emphasizes the importance of multidisciplinary monitoring and the urgent need for evidence-based guidelines to balance maternal benefits with fetal safety.

## 1. Introduction

Targeted temperature management (TTM) is a key neuroprotective strategy in patients with brain injury and cerebral edema. Its principal aim is to maintain normothermia to limit secondary brain injury and improve neurological outcomes [1,2,3]. Normothermia can reduce cerebral metabolic demand, limit excitotoxic neurotransmitter release, decrease oxidative stress, and modulate inflammatory response [1].

Applying TTM during pregnancy is challenging because maternal and fetus well-being are both sensitive to temperature and to the physiological effects of cooling. Maternal hyperthermia is associated with fetal risks, such as neural tube defects in the first trimester and fetal tachycardia or preterm labor at later stages [4]. Therapeutic hypothermia (32–34 °C) is generally avoided in pregnancy because fetal safety data are limited and hypothermia may impair uteroplacental vasoconstriction perfusion: therefore, maintaining normothermia (≈36–37.5 °C) is the preferred target in pregnant patients requiring neurocritical care [5,6,7,8]. The European Society of Intensive Care Medicine (ESICM) endorses continuous temperature control targeting normothermia, while recommending individualized clinician assessment in each case [9].

Available methods for temperature control range from antipyretics and conventional surface cooling to invasive endovascular devices. Conventional surface techniques can be difficult to titrate and may provoke shivering and temperature variability, while endovascular cooling, though precise, is invasive. The Arctic Sun surface temperature control system uses hydrogel pads and automated feedback regulation to provide precise, noninvasive control of core temperature, making it an attractive option when tight but noninvasive temperature control is needed.

We report the case of a pregnant woman with subdural empyema and severe cerebral edema in whom the Arctic Sun system was used to control refractory hyperpyrexia while balancing maternal neuroprotection with fetal monitoring.

## 2. Case Presentation

A 24-year-old pregnant woman, at 26 weeks of gestation with a history of splenectomy for corticosteroid-resistant thrombopenia, fully vaccinated and with an uncomplicated pregnancy till then, was admitted to the ICU after neurosurgical decompression for subdural empyema with severe cerebral edema. She arrived sedated, intubated, and mechanically ventilated.

Her illness had started nine days earlier (Day −9). That day, she visited a dentist for a toothache, and a dental abscess was diagnosed. Oral amoxicillin was prescribed. Five days later (Day −4), she developed a fever (38.5 °C) and headache and therefore visited the emergency department (ER) of a rural hospital. A physical examination revealed a fever (38.5 °C), cervical stiffness with meningism, face edema, and tachycardia (120 bpm). The ophthalmological evaluation excluded papilledema. A Lumbar puncture was compatible with central nervous system infection. Blood cultures were drawn and empiric antibiotics (ceftriaxone, vancomycin, metronidazole, acyclovir) were initiated. She was admitted to the ward. At that time, she remained hemodynamically stable with preserved consciousness (Glascow Coma Scale-GCS 15/15).

The day before her admission to our ICU (Day −1) her condition deteriorated. She developed seizures and a drop in consciousness (GCS 8/15). Brain computed tomography (CT) showed massive cerebral edema, midline shift, obliteration of subarachnoid spaces, and subdural empyema. She was intubated, and an intracranial pressure (ICP) monitoring catheter was inserted.

Over the following hours, ICP rose and fever persisted over 39 °C. Mannitol was administered and an (MRI) was performed. The MRI confirmed extensive subdural empyema, right lateral ventricle compression, and a purulent pararenal sinus infection.

Given this rapid deterioration, she was transferred to our tertiary hospital (Day 0). Neurosurgeons performed an extensive craniectomy, with empyema evacuation and the endoscopic drainage of paranasal sinuses by an otorhinolaryngologist. She was admitted postoperatively to the ICU deeply sedated (with midazolam, propofol, and remifentanil) and was ventilated with continuous ICP monitoring. She had a mild hemodynamic instability and was supported with low dose noradrenaline to target MAP of 65 mm Hg. Intravenous antibiotics (ceftriaxone, vancomycin, and metronidazole) were administered.

During the first ICU hours, she developed hyperpyrexia (>40 °C) and was unresponsive to pharmacologic therapy. For precise and sustained control, the Arctic Sun surface temperature management system was initiated. The target temperature was set to 37 °C, monitored with a bladder thermistor connected to the machine. The core temperature was successfully reduced to the target value in 3 h and maintained at that level, with continuous cooling operation for the next 3 days. Continuous electroencephalography (EEG) was used during this period to detect seizures, and ICP was monitored. Twice daily fetal assessment was performed with a cardiotocogram (NST), which revealed no evidence of fetal distress.

On ICCU Day 3, a spontaneous rupture of the follicle occurred. An emergency cesarean section was performed, and a premature live infant was delivered. The neonate was intubated and admitted to the neonate ICU (NICU), where intensive support continued.

**Figure 1:**
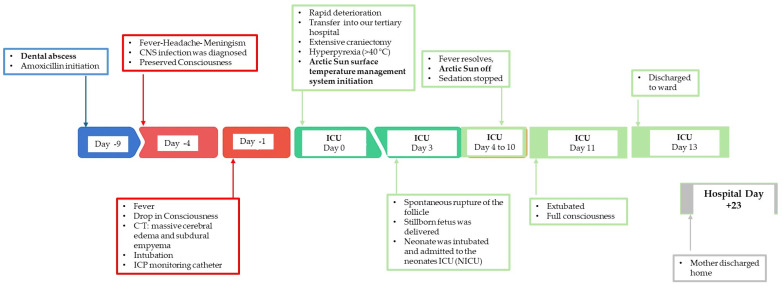
Schematic timeline of major events that took place during patient’s hospitalization.

In the following days (Days 4–10) the maternal fever subsided, sedation was discontinued, and the Arctic Sun system was withdrawn. Neurological function was improved, and on Day 11 she was extubated with a full recovery of consciousness. On Day 13, she was transferred to the neurosurgical ward and discharged home 10 days later without neurological deficit. A schematic timeline of events is illustrated in Figure 1.

The neonate was extubated 8 days later. Following a one-month NICU stay, the infant was discharged in good condition.

## 3. Discussion

The Arctic Sun system is a non-invasive, surface-based device for targeted temperature management (TTM). It uses adhesive hydrogel pads and servo-controlled chilled water to regulate core body temperature. Compared with intravascular cooling, it avoids the risks of a catheter-based technique. A 2011 study in comatose cardiac arrest survivors found no difference in outcomes between intravascular and surface cooling [10].

Evidence on TTM in pregnancy remains extremely limited and consists almost exclusively of case reports with heterogenous methods and mixed outcomes [6,11,12,13,14]. We identified five published cases of therapeutic hypothermia in pregnancy, all in the context of out-of-hospital cardiac arrest (Table 1). Our case represents the sixth and, uniquely, the first describing normothermia maintenance with the Arctic Sun system in a pregnant woman with cerebral edema due to central nervous system infection.

In our case, the mother tolerated TTM well, with stable intracranial pressures and successful fever control without hemodynamic deterioration. However, spontaneous pre-term delivery occurred on ICU Day 3, resulting in a premature but viable infant that ultimately managed to survive and was discharged home in excellent health status.

Whether premature delivery was related to TTM cannot be established. Potential contributing factors include maternal critical illness (severe intracranial infection, neurosurgery, and systemic inflammatory response), hyperpyrexia prior to TTM, and medication and sedation with multiple neuroprotective drugs and antiepileptics, which may have influenced fetal status. Of course, surface cooling could also have a role because, although effective, temperature shifts or shivering responses may theoretically alter uteroplacental blood flow. Thus, while the association with Arctic Sun cannot be excluded, it is more likely that multiple overlapping factors contributed to the preterm delivery.

The presented case reports, along with our presentation, highlight that the benefits of temperature control in critical maternal illness may outweigh the potential fetal risks, but only under close fetal and maternal monitoring. Across reported cases, maternal outcomes were consistently favorable, often with a full neurological recovery. All of them involved patients who were less than 24 weeks pregnant. Fetal outcomes were mixed: in four reports, pregnancies continued to term with healthy infants, while, in one, fetal demise occurred at 20 weeks. Our case differs in that it involves neuroprotection rather than post-arrest indication for TTM, a longer gestation (26 weeks), and the use of controlled normothermia rather than hypothermia. Collectively, these cases suggest that TTM can be safely considered in pregnancy when maternal survival and neurological protection are at stake but underscore the need for vigilant fetal monitoring and individualized decisions. In this respect, further investigation is needed. Each decision must involve a multidisciplinary team (critical care, neurosurgery, obstetrics), weigh risks and benefits, and prioritize maternal survival while continuously monitoring the fetus.

Now, international societies adopt a targeted temperature management maintaining normothermia instead of hypothermia [7,8], and, in this aspect, pregnancy was recently included [15].

Our report, like the prior literature, is limited by its single-patient nature. No large-scale data exist to guide TTM use in pregnant women, and Randomized Control Trials (RCTs) beyond case reports are needed.

All case reports presented here involved pregnant women who suffered from cardiac arrest with temperature management being a component of post-resuscitation care. This is the first report of Arctic Sun-guided TTM for cerebral edema due to central nervous system infection in pregnancy (neuroprotection) in the light of a French expert panel that recommends targeted temperature management for controlling raised intracranial pressures [16].

In our case, the use of the Arctic Sun temperature control system appeared to be both safe and effective for the mother, but whether the targeted temperature management contributed to the premature delivery of the fetus cannot be excluded.

## 4. Clinical Pearls

• Pregnancy is not an absolute contraindication to TTM. Our case supports the cautious use of surface cooling with Arctic Sun under continuous maternal and fetal monitoring.
• Multidisciplinary decision-making is essential. Optimal care requires coordinated input from intensivists, neurosurgeons, obstetricians, neonatologists, and nursing staff.
• Maternal survival should be balanced with fetal safety. Maternal stabilization and neuroprotection are priorities, but decisions must also incorporate gestational age, fetal monitoring data, and potential risks of cooling.
• Fetal outcomes remain uncertain. Previous reports show both favorable and adverse fetal results, with stillbirths occurring in some but not all cases.
• Research gap: The current evidence is limited to isolated case reports. Systematic data collection, registries, or multicenter collaborations are urgently needed to clarify safety and outcomes.
• Clinical takeaway: When maternal neurological injury is life-threatening, advanced temperature management can be justified in pregnancy, provided that monitoring and individualized risk–benefit assessments are rigorously applied.

## 5. Conclusions

This case demonstrates that targeted temperature management with the Arctic Sun system can be safely applied in critically ill pregnant patients, even outside of the post-cardiac arrest setting. While maternal outcomes were favorable, the premature fetus delivery underscores the uncertainty regarding fetal safety and highlights the need for multidisciplinary decision-making. Systematic data collection is required to better define risks, benefits, and optimal protocols for temperature control in pregnancy.

## Figures and Tables

**Table 1:** Summary of published case reports of TTM in pregnancy.

Author (Year)	Maternal Condition	Gestational Age	Cooling Method	Target Temperature and Duration	Maternal Outcome	Fetal Outcome
Rittenberger et al. (2008) [13]	Out-of-hospital cardiac arrest (VF)	13 weeks	Cold saline + ice packs → intravascular catheter	33 °C for 24 h	Discharged with mild neurological deficit	Term delivery (39 week), normal infant neurodevelopment
Wible et al. (2010) [11]	Out-of-hospital cardiac arrest (VF)	20 weeks	Cold saline + Arctic Sun	33 °C for 24 h	Good neurologic recovery	Fetal demise (20 weeks)
Chauhan et al. (2012) [6]	Out-of-hospital cardiac arrest	20 weeks	Arctic Sun surface system	32.6 °C for 24 h	Full neurologic recovery	Term delivery (39 week), normal infant, normal 3-year follow-up
Oguayo et al. (2015) [12]	Out-of-hospital cardiac arrest	18 weeks	Arctic Sun surface system	33 °C for 24 h	Complete neurologic recovery	Term delivery (40 week), healthy infant
Hogg et al. (2020) [14]	Out-of-hospital cardiac arrest (maternal arrhythmia)	2nd trimester (exact gestational age not stated)	Cooling protocol (method not specified)	Not reported	Full recovery	Term delivery (39 week), healthy infant
Present case (2025)	Cerebral edema from subdural empyema	26 weeks	Arctic Sun surface system	Normothermia maintenance (37 °C)	Excellent neurological recovery, extubated day 11, discharged	Preterm cesarean delivery after spontaneous rupture; infant in neonate ICU, discharged healthy

## Data Availability

The original contributions presented in this study are included in the article. Further inquiries can be directed to the corresponding author.
